# Response Preparation and the Simon Effect: Experimental and Model-Based Analyses

**DOI:** 10.5334/joc.471

**Published:** 2026-01-07

**Authors:** Herbert Heuer, Peter Wühr

**Affiliations:** 1 Leibniz Research Center for Working Environment and Human Factors (IfADo), Germany; 2 TU Dortmund University, Germany

**Keywords:** leaky, competing accumulator model, monitor distance, response frequency, response preparation, response cues

## Abstract

In choice between frequent and infrequent responses the Simon effect is larger for the frequent than for the infrequent response. We arbitrate between three hypotheses to account for this finding. The first hypothesis holds that it is a straightforward consequence of biased response preparation. The second hypothesis posits a facilitation of the shift of visual attention to the side of the prepared response in addition, and the third one an effect of the different frequencies of congruent and incongruent trials associated with the task-irrelevant stimulus locations. In three experiments we show the modulation of the Simon effect by relative response frequency, its independence from the distance between hands and monitor, and its almost complete elimination by valid response cues. These findings are in line with a primary role of biased response preparation. Consistent with this conclusion, in a model-based analysis, using extensions of the Leaky, Competing Accumulator model, differences between the probabilities of preparing the frequent and infrequent response were sufficient to produce the modulation of the Simon effect, though only poorly its dynamics as assessed by delta plots. However, these dynamics were produced by a model which implemented the hypothesis that response preparation implicates shielding against distraction in addition to anticipatory response activation. According to simulation results, the modulation of congruency effects by relative response frequency might depend on the particular type of congruency effect, specifically the temporal offset between the impacts of relevant and irrelevant stimuli.

## 1. Introduction

Effective task performance requires not only the processing of task-relevant, but also the neglect of task-irrelevant information. However, often the neglect of task-irrelevant information is less than perfect. Experimentally this is studied by means of conflict tasks. Prominent among them is the Simon task where a stimulus feature such as color defines the correct response location, whereas the stimulus location, which can be congruent or incongruent with the response location, is task-irrelevant. Nevertheless, responses are faster and more accurate with congruent than with incongruent stimulus and response locations. This is a particular instance of a congruency effect, also known as Simon effect. Previously we have observed that it is stronger for responses with larger relative frequency than for responses with smaller relative frequency. Here we contrast three different hypotheses to account for this modulation of the Simon effect both experimentally and by comparing the predictive power of extensions of the Leaky, Competing Accumulator (LCA) model which implement these hypotheses.

### 1.1. Congruency effects in the Simon task

In a typical Simon task, named after the seminal study of Simon and colleagues (e.g., Simon, 1969; Simon & Rudell, 1967), participants respond rapidly and accurately to a nonspatial stimulus feature such as the pitch of a tone or the color of a visual stimulus by pressing a left or right key. This relevant stimulus feature varies randomly from trial to trial, and so does the stimulus location, a task-irrelevant stimulus feature, which is to the left or right of a central fixation mark. In congruent trials the response location is the same as the stimulus location (left-left, right-right), but in incongruent trials the two locations differ (left-right, right-left). Reaction times are shorter and accuracy is higher in congruent than in incongruent trials (reviews: Lu & Proctor, 1995; Cespón, Hommel, Korsch, & Galashan, 2020). The prevalent account of the Simon effect is in terms of a dual-route model where response codes are activated both by a conditional or controlled route (with the relevant information as source) and an unconditional or automatic route (with the irrelevant information as source) (Kornblum, Hasbroucq, & Osman, 1990).

The Simon effect, as other congruency effects, varies across the range of reaction times. These dynamics of congruency effects are studied by means of delta plots which were introduced by De Jong, Liang, and Lauber (1994). Delta plots show the mean differences between incongruent and congruent conditions (and thus, the Simon effects) across quantiles of the reaction-time distributions. More specifically, the differences between the pairs of quantiles in the two conditions are plotted against the means of these pairs (Speckman, Rouder, Morey, & Pratte, 2008). Depending on other experimental conditions, delta plots can adopt various shapes (Proctor, Miles, & Baroni, 2011). Whereas for most conflict tasks congruency effects increase as reaction times become longer, for the (visual) Simon task with horizontal stimuli and responses they mostly decline (Burle, Possamaï, Vidal, Bonnet, & Hasbroucq, 2002; Burle, Spieser, Servant, & Hasbroucq, 2014; Mittelstädt, Miller, Leuthold, Mackenzie, & Ulrich, 2022; Pratte, Rouder, Morey, & Feng, 2010; Wiegand & Wascher, 2005). Increasing delta plots are consistent with the general rule that reaction-time variability increases with the mean (Wagenmakers & Brown, 2007), but declining delta plots violate this rule (Zhang & Kornblum, 1997) and thus are “special”. A rather popular account of the decline posits inhibition of the influence of the irrelevant stimulus feature in the course of response selection (Van den Wildenberg, Wylie, Forstmann, Burle, Hasbroucq, & Ridderinkhof, 2010).

### 1.2. Relative response frequency and the Simon effect

The effect of relative stimulus-response (S-R) frequency is well established: the more frequent S-R pair has shorter reaction times and higher accuracy than the less frequent one (Fitts, Peterson, & Wolpe, 1963; Teichner & Krebs, 1974). The superior performance with the more frequent S-R pair is largely based on correct expectations in the majority of trials and the associated response preparations (Miller & Anbar, 1981; Miller, 1998). When participants are asked to verbalize their expectations, the relative frequencies of expecting the one or the other of the two S-R alternatives roughly match their objective relative frequencies, and reaction times are faster for the expected S-R pairs than for the unexpected ones (Bernstein & Reese, 1965; Hinrichs, 1970; Hinrichs & Craft, 1971).

The relative frequency of the two S-R alternatives in a binary choice task does not only affect reaction time and accuracy, but also the size of the Simon effect: it is smaller for the infrequent response than for the frequent one, even when stimuli have identical frequencies as when three stimuli are mapped to one of the two responses and only one stimulus to the other one (Wühr & Heuer, 2015). Thus, it is the relative frequency of responses rather than of stimuli which is critical for the modulation of the Simon effect. Regarding the dynamics of the Simon effect for frequent and infrequent responses, for frequent responses the effect tended to increase with increasing reaction times, but with infrequent responses it tended to decrease.

At first glance, the reduced Simon effect observed with the longer reaction times of infrequent responses may appear counterintuitive because with longer reaction times there should be more time for the influence of the task-irrelevant stimulus location. On the other hand, a decrease of the Simon effect for longer reaction times could be expected from the observation of declining delta plots (Proctor & Wang, 1997). This account implies that delta plots for frequent and infrequent responses are basically the same, covering only different ranges of reaction times. However, these delta plots indicate different congruency effects even at identical reaction times and tend to diverge, as described above (cf. Wühr & Heuer, 2015).

Here we contrast three hypotheses to account for the modulation of the Simon effect by relative response frequency. Two of them ascribe the modulation to the different expectations (and preparations) of the frequent and infrequent responses rather than to the different relative frequencies per se. These two hypotheses are motivated by the evidence of the modulation of the Simon effect not only by different relative response frequencies, but also by valid and invalid response cues: the Simon effect is stronger with valid than with invalid cues (Proctor, Lu, & Van Zandt, 1992; Verfaellie, Bowers, & Heilman, 1988; Wascher & Wolber, 2004; Wühr, 2006). The third hypothesis ascribes a critical role to the relative response frequencies which produce contingencies between task-irrelevant stimulus locations and congruency.

**Figure 1:**
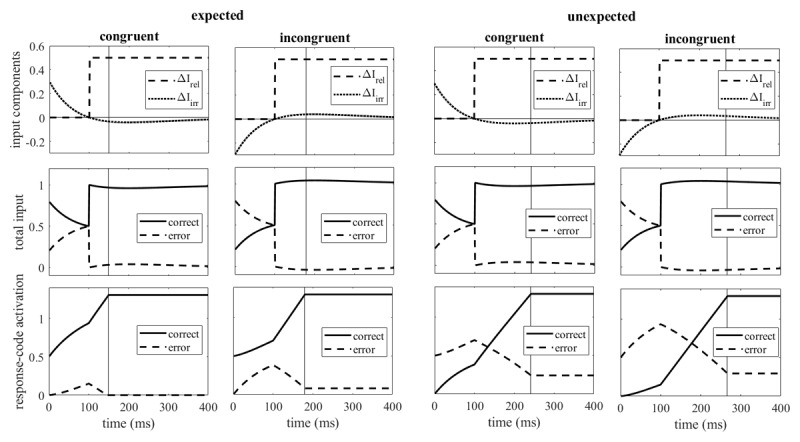
Components of the external incremental input (top row), total external input (middle row), and response-code activation (bottom row) for both expected and unexpected congruent and incongruent trials. The vertical lines mark the time when the threshold is reached. (Detailed explanation in text).

The first and most basic hypothesis is the *preparation hypothesis*. It holds that the modulations of the Simon effect by response-cue validity and by relative response frequency have the same cause, namely biased expectations and preparations of responses. This hypothesis is a kind of default that remains as other hypotheses can be excluded: The evidence of biased expectations and preparations of responses with different relative frequencies is quite robust and without doubt, but it is not intuitively obvious how these should result in different Simon effects. Possibly the biased response preparations modulate the effects of the irrelevant stimulus locations on the activations of the two responses. We expect that the results of our modelling will produce further insights on this issue (see General Discussion). As an example, Figure 1 illustrates how a simple difference in response preparation can produce the modulation of the Simon effect in principle.

The second hypothesis is the *visual-attention hypothesis* which posits that biased preparations are accompanied by biased shifts of visual attention. It is based on a finding of Wascher and Wolber (2004): in response-cueing experiments, the cues (arrows pointing to the left or right) accelerated the shift of visual attention to the stimulus location on the cued side relative to that on the non-cued side as indicated by the latency of an electrophysiological indicator (N2pc, PCN). Wascher and Wolber hypothesized that these attentional differences give rise to differences in the processing of the relevant stimulus feature and thereby reaction times. Specifically, responding in congruent trials should be facilitated for validly cued responses, thereby increasing the Simon effect, whereas responding in incongruent trials should be facilitated with invalid cues, thereby reducing the Simon effect.

Although Wascher and Wolber (2004) provided convincing evidence for cue-related different latencies of the shift of visual attention to the stimulus location for spatial response cues, as they were typically used (Proctor et al., 1992; Verfaellie et al., 1988; Wühr, 2006), one may doubt that such differences are also present when expectations of the one or the other response are induced by different relative frequencies. Even in response-cueing experiments the different latencies of the shifts of visual attention are not a robust phenomenon. For example, Buhlmann and Wascher (2006) used non-spatial response cues (color) and still found the modulation of the Simon effect, but not the different latencies of the N2pc. However, they observed different amplitudes of the N2pc which also might reflect attentional differences.

The third hypothesis is the *contingency hypothesis*. When the relative frequencies of the two responses in a standard Simon task are different, contingencies between irrelevant stimulus locations and congruency are a by-product.^1^ This is detailed in Table 1 for the case where the left response is the frequent one. Provided that both frequent (75% of all trials) and infrequent responses (25% of all trials) are congruent and incongruent with identical relative frequencies of 50%, for the left stimulus location 75% of the responses would be congruent and only 25% would be incongruent, whereas for the right stimulus location 75% of responses would be incongruent and only 25% of responses would be congruent. This difference in the proportion of congruent trials could result in location-dependent effects of the irrelevant stimulus location, that is, in context- or location-specific congruency effects: with a higher proportion of congruent trials at a certain location congruency effects are typically larger than with a smaller proportion (e.g., Hübner & Mishra, 2016; Crump, Gong, and Milliken, 2006, for the Stroop effect).

**Table 1:** Contingencies between irrelevant stimulus locations and congruency, when one response is more frequent than the other.


	LEFT STIMULUS LOCATION (50%)	RIGHT STIMULUS LOCATION (50%)

Left Response (frequent: 75%)	37.5% of trials condition frequent-congruent	37.5% of trials condition frequent-incongruent

Right Response (infrequent: 25%)	12.5% of trials condition infrequent-incongruent	12.5% of trials condition infrequent-congruent

effect of irrelevant stimulus location	mostly congruent (75:25%): stronger effect	mostly incongruent (75:25%): weaker effect


In the present experiments the situation is complicated by the fact that for each response congruent and incongruent conditions are associated with different irrelevant stimulus locations and thus different location-specific proportions of congruent trials. As is evident from Table 1, congruent high-frequency responses are associated with a location with a high proportion of congruent responses and should be facilitated, whereas incongruent high-frequency responses are associated with a location with a high proportion of incongruent responses, which should reduce interference from the incongruent stimulus location. In contrast, congruent low-frequency responses are associated with a stimulus location with a high proportion of incongruent responses, which should reduce facilitation, whereas incongruent low-frequency responses are associated with a stimulus location with a high proportion of congruent responses, which should increase interference from the irrelevant stimulus location. Crucially, for the frequent responses the stronger facilitating influence of congruent stimulus locations and the weaker inhibitory influence of incongruent stimulus locations as compared with infrequent responses could compensate each other as far as the Simon effect with frequent and infrequent responses is concerned. However, the compensation could also be imperfect. For example, if the difference between the frequent and the infrequent response in facilitation from congruent locations is stronger than the difference in inhibition from incongruent locations, then the different contingencies could increase the Simon effect for the frequent relative to the infrequent response.

We tested the three hypotheses both experimentally and by model-based analyses. After demonstrating the modulation of the Simon effect by relative response frequency in Experiment 1, in Experiment 2 we contrasted the preparation and visual-attention hypotheses by varying the distance between the hands and the monitor on which the stimuli were presented, and in Experiment 3 we contrasted the preparation and contingency hypotheses by adding response cues so that frequent and infrequent responses could be expected equally, whereas the contingencies of irrelevant stimulus locations and congruency were unaffected. For the model-based analysis we used extensions of a sequential-sampling model which has served previously to account for congruency effects (Wühr & Heuer, 2018; Heuer, Seegelke, & Wühr, 2023; Heuer & Wühr, 2025; Wühr & Heuer, 2025a). In the following we shall detail both the experimental and the model-based approaches to evaluating the hypotheses.

### 1.3. Expectations and attention shifts

The visual-attention hypothesis holds that the expected and prepared left or right response facilitates the shift of visual attention to the spatially corresponding location on the monitor. A facilitation of lateralized attention, inferred from a shortened latency of the N2pc, has been observed by Wascher and Wolber (2004) when spatial response cues were presented on the monitor, but with symbolic cues Buhlmann and Wascher (2006) found only an amplitude difference of the N2pc between valid and invalid cues. When response preparation is varied by means of relative response frequency, there are no visual cues at all. One may doubt that under such conditions preparation of a response is also accompanied by a facilitated shift of visual attention to the corresponding location on the monitor. Even if facilitation was present with a monitor close to the hands, according to two lines of evidence it should disappear – and so should the modulation of the Simon effect by relative response frequency – when the distance between the attended hand and the monitor is increased.

The first line of evidence is from the nearby-hands effect: visual processing is facilitated when a hand of the observer is in the vicinity of the monitor where the visual stimuli are presented (Tseng, Bridgeman, & Juan, 2012, for review). Crucially, this nearby-hands effect vanishes as the distance between hands and stimuli is increased. The second line of evidence is from studies of cross-modal attention: the cross-modal effect of attending to a stimulus in one modality on attention for a stimulus in a second modality depends on distance. Specifically, it is reduced when the distance between the attended hand and visual stimuli is increased (e.g., Eimer & Driver, 2001).

Both the nearby-hands effect and spatially constrained haptic to visual cross-modal attention are likely related to a certain type of bimodal neurons (cf. Eimer & Driver, 2000; Tseng et al, 2012). These are neurons with both tactile and visual receptive fields, with the visual fields covering a region surrounding the bodily surface where the tactile fields are located (e.g. Graziano & Gross, 1993; for humans see Makin, Holmes, & Zohary, 2007). In fact, visuo-tactile integration has become a kind of hallmark of peripersonal space (Clery, Guipponi, Wardak, & Hamed, 2015; Ladavas, di Pellegrino, Farne, & Zeloni, 1998; Maravita, Spence, & Driver, 2003) which surrounds the human body. A distant monitor outside peripersonal space should thus abolish the relation (or coupling) between an attentional focus on the hand for the prepared response and the shift of visual attention to the corresponding side of the monitor. Consequently, under the visual-attention hypothesis the modulation of the Simon effect by relative response frequency should be abolished as well. This prediction was tested in Experiment 2.

### 1.4. Expectations and contingencies

When relative response frequencies in a binary choice task are different, the more frequent responses are expected and prepared more frequently than the less frequent responses (Bernstein & Reese, 1965; Hinrichs, 1970; Hinrichs & Craft, 1971; Miller, 1998). This difference can be nullified with consistently valid response cues. Even when participants do not always make use of the information provided by the cues, the difference between frequent and infrequent responses with respect to preparation should be essentially absent. However, the different contingencies of irrelevant stimulus locations and congruency persist. Thus, a persistent modulation of the Simon effect by relative response frequency would provide support for the contingency hypothesis, whereas its absence (or strong reduction) would strengthen the preparation hypothesis. These predictions of the hypotheses were tested in Experiment 3.

With consistently valid response cues the Simon task can in principle be turned into a simple reaction-time task: given the valid response cue, there remains only temporal uncertainty, but no response uncertainty. If this were the case, the Simon effect should disappear, no matter whether responses are frequent or infrequent. However, even with consistently valid response cues the Simon effect is still present, though reduced in size as compared with a control condition without response cues (Wühr, 2006). Similarly, the flanker effect, a different congruency effect, is not reliably abolished by consistently valid response cues (Wühr & Heuer, 2017; Wühr, Frings, & Heuer, 2018). Perhaps the persistence of congruency effects in spite of advance knowledge of the forthcoming response is related to the fact that with consistently valid response cues the task becomes a simple reaction-time task from the perspective of a single trial, but not from the perspective of a series of trials which call for different responses. Thus, on a longer time scale the tasks remain choice tasks, and a task-set comprising two S-R mappings might be preferred over frequent switches between task sets for simple reactions. This, of course, is likely to change with longer series of trials requiring the same response (cf. Hommel, 1996).

### 1.5. Model-based analysis of the hypotheses

We use extensions of the Leaky, Competing Accumulator (LCA) model, which has been introduced by Usher and McClelland (2001), to test whether the modulation of the Simon effect by relative response frequency could be a straightforward consequence of biased response preparation or requires additional processing variations as posited by the visual-attention or the contingency hypotheses. Here we describe the basics of the LCA model and of our extensions. Details are provided in Appendix A.

For a binary decision the alternative responses are represented by two response codes, one for correct responses and one for errors. The response codes are activated until one of them reaches a threshold. Incremental activation per unit of time is the sum of external stimulus-related inputs, self-inhibition, lateral inhibition, and noise. Self-inhibition shapes the negative acceleration of response-code activation and its final asymptote, lateral inhibition increases the activation difference between the two response codes, and noise produces errors and variability of the number of units of time until the threshold is reached. A residual time is added to the time required for reaching the threshold, to take the durations of stimulus identification and response production into account, as is the case for all sequential-sampling models (cf. Forstmann, Ratcliff, & Wagenmakers, 2016).

In the LCA model, as developed by Usher and McClelland (2001), the external stimulus-related input to the correct response code is time-invariant. The external input to the incorrect response code is one minus the external input to the correct response code (Usher & McClelland, 2001, p. 559). This constraint on the sum of external inputs to both response codes amounts to defining a scale for a subset of model parameters, as it is required by essentially all sequential-sampling models (cf. Donkin, Brown, & Heathcote, 2009).

To account for congruency effects, we made additions to the external stimulus-related input. Consistent with the dual-route model and similar to a computational model of Zorzi and Umilta (1995) and the diffusion model for conflict tasks by Ulrich, Schröter, Leuthold, and Birngruber (2015), we added to the time-invariant “controlled input” from the task-relevant stimulus feature a time-varying “automatic input” from the task-irrelevant stimulus feature. For the automatic (or irrelevant) input we assumed a decline with the passage of time and a variable temporal offset from the controlled (or relevant) input (Wühr & Heuer, 2018; Heuer, Seegelke, & Wühr, 2023; Heuer & Wühr, 2025). For example, in the Simon task processing of the (task-irrelevant) stimulus location leads the processing of the task-relevant stimulus color (Cespon et al., 2020; Zorzi & Umilta, 1995), whereas processing of the task-irrelevant stimulus size, which also affects choice between left-hand and right-hand responses (e.g. Wühr & Seegelke, 2018; Richter & Wühr, 2022, 2023, 2024), lags the processing of the task-relevant stimulus color. These different temporal relations result in characteristically different delta plots (Heuer et al., 2023; Wühr & Heuer, 2025a).

In previous applications of the model to conflict tasks we assumed an exponential decline of the automatic input: starting at an initial value above zero in congruent trials and below zero in incongruent trials it approached zero without changing its arithmetic sign, that is, without switching from facilitation to inhibition or vice versa. This differs from the dynamics of the automatic input of the diffusion model for conflict tasks of Ulrich et al. (2015; see their Figure B1, lower graph), which allows a change of the arithmetic sign. Although we had argued that the assumption of such a change lacks plausibility (Heuer et al., 2023; see also Lee & Sewell, 2024, Janczyk, Mackenzie, & Koob, 2025), we revised this view after a model-based analysis of the modulation of the Simon effect by the congruency of the preceding trial, known as congruency sequence effect (Heuer & Wühr, 2025). In fact, when comparing a model with exponential decline and a model with a decline that allows a temporary switch of the arithmetic sign, we found a better fit of the latter type of model (Wühr & Heuer, 2025a). Therefore, for the present simulations we have replaced the exponential decline, which corresponds to the step response of a first-order high-pass filter, by the step response of a second-order high-pass filter (see Figure 1 for illustration and Appendix A for the equation).

Here we extend the model further to account for the effects of relative response frequency, specifically the bias toward the more frequent response. The most obvious parameter of sequential-sampling models to represent this bias is the initial state of the decision process (cf. Voss, Rothermund, & Voss, 2004; Wühr & Heuer, 2017). For the diffusion model Ratcliff and McKoon (2008) indeed found that most of the effect of relative S-R frequency could be accounted for by a biased initial state. However, there was also a small contribution from a difference in drift rates, that is, the speed of processing the frequent and the infrequent stimulus. Such a difference, however, is unlikely for the present experiments in which stimuli were equally frequent and relative response frequency was varied by mapping three stimulus colors to one of the two responses and only one color to the other response. In fact, preliminary analyses indicated no substantial improvement of the fit of the present models by allowing the controlled (relevant) input to be different for the frequent and infrequent response.

Figure 1 illustrates the time course of the components of the external input as well as the total external input and the response-code activations. The illustrations are for a lead of the irrelevant input (by 100 ms), as it is typical for the Simon effect. The irrelevant input (ΔI_irr_ in the top row of Figure 1) starts at positive values in congruent trials and at negative values in incongruent trials. Note that it crosses zero (after 100 ms in the example) and thus changes its arithmetic sign before it reaches zero. Both the relevant and the irrelevant external input (and thus the total external input) are the same for expected and unexpected responses. The effect of expectation is limited to the initial activation levels of the response codes (bottom row of Figure 1): when the correct response is expected, its initial activation is larger than that of the error response, but when the correct response is unexpected (and the error response is the expected one), the initial activation of the error response is larger than that of the correct response. In the example of Figure 1 the differences between incongruent and congruent trials are 30 ms for expected responses and 24 ms for unexpected responses, which qualitatively corresponds to the observed modulation of the Simon effect.

For modeling the results of Experiments 1 and 2, we assumed that the more frequent response is expected in a certain proportion π of trials and the less frequent response in the remaining proportion 1-π of trials. This ‘preparation model’ implements the *preparation hypothesis*: frequent and infrequent responses differ only in the proportion of trials in which they are correctly expected and prepared. Of course, with the response cues in Experiment 3 the proportions of expected frequent and infrequent responses no longer add to 1. Instead both should be expected and prepared in almost all trials, that is, with values of π_freq_ and π_infr_ close to 1, though possibly different.

The preparation model was extended to implement the visual-attention and the contingency hypotheses. For the *visual-attention hypothesis* we allowed different relevant inputs when stimuli were presented on the same or opposite side (left vs right) as the expected response: processing of the stimulus on the same side should be facilitated as compared to processing of the stimulus on the opposite side. For frequent responses facilitated processing would be more frequent in congruent than in incongruent trials, and for infrequent responses it would be more frequent in incongruent than in congruent trials. This +attention model, where the + indicates that there is an addition to the preparation model, was not fit to the data of Experiment 3 because with always valid response cues the relative frequency of preparing the correct response should be close to 1 both for frequent and infrequent responses. Without a difference in the frequency of correct expectations any related difference in the shifts of visual attention and the associated stimulus processing should be absent as well.

As implementation of the *contingency hypothesis* in the ‘+contingency model’ we allowed both different relevant and irrelevant inputs when stimuli were presented at the different task-irrelevant stimulus locations, that is, for conditions frequent-congruent and infrequent-incongruent on the one hand and frequent-incongruent and infrequent-congruent on the other hand. The reason to allow both parameters to differ is that the influence of an irrelevant stimulus feature on reaction time can in principle be reduced by increasing the relative impact of the relevant feature or reducing the relative impact of the irrelevant feature. For the effects of the proportion of congruent trials it is not yet clear, to our knowledge, whether the one, the other, or both parameters are affected.

## 2. Method

### 2.1. Participants

We collected data from 56 participants in three experiments. The 16 participants (14 female, 2 male) of Experiment 1 were, on average, 21.81 years of age and mostly right-handed (15). The 16 participants (15 female, 1 male) of Experiment 2 were, on average, 24.69 years of age and mostly (15) right-handed. Finally, the 24 participants (19 female, 5 male) of Experiment 3 were, on average, 23.13 years of age and mostly (22) right-handed. All participants gave their written informed consent before participating in the experiment. There were no particular criteria for inclusion in our study except that age should be between 18 and 40. Participants received course credits for participating in our experiments.

Experiment 1 is a re-analysis of the data from Experiment 2 of Wühr and Heuer (2015).^2^ As reported below, in this data set the effect size of the difference between the Simon effects with frequent and infrequent responses, that is, the effect size of the modulation of the Simon effect, was *d* = 1.076. With an effect size of 1, a sample size of 13 is required for a t-test with α of .05 and power of .9 according to GPower (Faul, Erdfelder, Lang, & Buchner, 2007). Therefore, we ran Experiment 2 with the same number of 16 participants as Experiment 1, although here the interest was primarily in the difference between the modulations of the Simon effect by the different monitor distances. For a one-sided t-test of this difference with n = 16, α = .05 and (1-β) = .9, the critical population effect size is *d* = 0.767. This effect size is in the expected range for the difference between the modulations observed with the near and distant monitor if the modulation were almost absent with the distant one. In Experiment 3 there were 24 participants, the number required for *d* = 0.7, α = .05, and 1-β = .9, because in Experiment 2 the effect size of the modulation of the Simon effect was only *d* = 0.686.

### 2.2. Apparatus

Participants sat at a table and faced a monitor. In Experiments 1 and 3 the monitor was placed at a distance of about 50 cm from the participant, whereas in Experiment 2 it was placed exactly at 50 cm or 150 cm distance from the edge of the table in separate conditions (and sessions), with the order of distances balanced across participants. Responses were keypresses with the left hand (on the “Control” key at the lower-left corner of the keyboard) and with the right hand (on the “Enter” key at the lower-right corner of the keyboard).

### 2.3. Design and procedure

In all three experiments, the imperative stimulus was a filled square (1.5 cm × 1.5 cm in Experiments 1 and 3; 2 cm × 2 cm in Experiment 2) in one of four possible colors, presented to the left or right of a central fixation mark (plus sign, Arial font size 24). The four colors were blue (RGB = 0, 176, 240), green (RGB = 0, 176, 80), red (RGB = 255, 0, 0), and yellow RGB = (255, 255, 0). The horizontal distance between the imperative stimulus and the fixation mark (i.e., screen center) was 4 cm in Experiments 1 and 3, and 14 cm in Experiment 2. The response cues in Experiment 3 were centrally presented words “links” (left) and “rechts” (right) in Arial font with size 24.

One of the four stimulus colors, which was systematically varied across participants, was mapped to one response and the other three colors were mapped to the other response. Thus, the relative response frequencies were 75 and 25%, but the relative stimulus frequencies were all 25%. For half of the participants the frequent response was the right one, and for the other half it was the left one. Whereas color was the relevant stimulus feature, stimulus location, which was to the left or right of the fixation mark with equal relative frequencies, was the irrelevant stimulus feature.

In Experiment 1 there were two practice blocks with 24 trials each and 10 test blocks with 48 trials each. The experimental trials had a constant duration of 2,500 ms. After a blank period of 500 ms, the fixation point was presented for 1,000 ms. Five hundred milliseconds after the onset of the fixation point the imperative stimulus was added and remained on for 500 ms. Thus, fixation point and imperative stimulus disappeared simultaneously, followed by a blank period of 1,000 ms. Beginning with the onset of the imperative stimulus, the computer registered keypresses and measured RT for a period of 1,500 ms. If the response was correct, the next trial started immediately. If the response was incorrect, or if RT exceeded 1,000 ms, a corresponding error message was shown for two seconds.

Experiment 2 consisted of two sessions on separate days. Each session included a practice block of 32 trials, and 10 test blocks of 32 trials each. The two sessions only differed with regard to the distance between the participant and the monitor. In one session, the distance was 50 cm (as in Experiments 1 and 3), whereas in the other session it was 150 cm. The experimental trials had a variable duration of 2,500 ms + RT. After a blank period of 1,000 ms, the fixation point was presented for 500 ms. Next, the imperative stimulus appeared and the display remained until participants made a response, or until 2,000 ms had elapsed without response. If the response was correct, a blank screen followed the response for 1,000 ms. If, however, the response was incorrect, or if no response had been made in the presence of the imperative stimulus, a corresponding error message was shown for one second.

In Experiment 3 there were two practice blocks and eight test blocks of 48 trials each. A blank screen of 1,000 ms was replaced by the response cue for 500 ms, followed by the fixation mark for a variable cueing interval of 300, 500 or 700 ms which served to introduce temporal uncertainty and thereby reducing the number of anticipatory responses. The imperative stimulus was added for another 500 ms, followed by a blank screen for 1,000 ms. Feedback was provided when responses were anticipatory (before presentation of the imperative stimulus), or reaction times were longer than 1,500 ms. The total number of trials analyzed per participant was 480 in Experiment 1, 640 in Experiment 2, and 384 in Experiment 3.

### 2.4. Analyses

For each experiment trials were sorted into four conditions: frequent vs infrequent and congruent vs incongruent. For Experiment 2 we ran initial analyses including the effects of the two monitor distances, but such effects turned out to be absent. Therefore, the data for the two distances were collapsed for further analyses.

For each participant there were 180 trials in the two frequent and 60 trials in the two infrequent conditions each of Experiment 1; in Experiment 2 there were 240 and 80 trials, and in Experiment 3 there were 144 and 48 trials. For each of the four conditions trials were screened for outliers, defined by reaction times less than 100 ms or longer than mean plus three standard deviations. For each participant and experimental condition, the error percentage (after discarding outliers) and the mean reaction time of correct responses were determined. For reaction times of correct responses, we also computed five quantiles (.1, .3, .5, .7, .9) of their distribution in each condition. From these quantiles we computed delta plots which show the differences between quantiles in incongruent and congruent conditions as a function of the means of the quantiles in the two conditions (cf. Speckman et al., 2008).

Mean reaction times were subjected to two-way within-participant ANOVAs with the factors relative frequency and congruency; only for the preliminary analysis of Experiment 2 distance of the monitor was added as a third factor. We also compared the Simon effects with frequent and infrequent responses by means of t-tests for each experiment. Error percentages were analyzed by means of non-parametric Wilcoxon signed-rank tests because of substantial deviations from normal distributions according to Shapiro-Wilk tests. Delta plots were quantified by coefficients of orthogonal polynomials up to second order; for each individual delta plot the orthogonal polynomials were computed according to Emerson (1982), and their coefficients were computed according to Draper and Smith (1966, p.151). The coefficients were compared between the two relative response frequencies by means of t-tests.

We supplemented the frequentist analyses by Bayesian analyses and report Bayes factors in favor of the alternative hypothesis (B_1_ > 1) or the null hypothesis (B_0_ > 1). Following the classification described by Wagenmakers et al. (2018), we interpret Bayes factors <3 as ‘anecdotal evidence’, between 3 and 10 as ‘moderate evidence’, between 10 and 30 as ‘strong evidence’, between 30 and 100 as ‘very strong evidence’, and >100 as ‘extreme evidence’. All statistical analyses were done with JASP 0.18.3.

For the model-based analysis we pooled the reaction times of all participants for each of the four conditions of each experiment after linear transformations as described by Sternberg (2024). For each participant *i* the reaction time *x_ij_* in trial *j* in any of the four conditions was transformed into \[{{y}_{ij}}={{m}_{.}}+\left({{x}_{ij}}-{{m}_{i}}\right)\frac{{{q}_{.}}}{{{q}_{i}}}\], with *m_i_* as the mean of participant *i* and \[{{m}_{.}}\] as the mean of these individual means. Similarly, *q_i_* is a robust scale estimator for participant *i* and \[{{q}_{.}}\] is the mean of the individual scale estimators. The scale estimator is based on an order statistic of the absolute differences between observations (Rousseeuw & Croux, 1993; Croux & Rousseeuw, 1992). According to Sternberg (2024), this method of combining individual RT distributions is more accurate in estimating the average of the individual distributions than other and more established methods such as vincentizing (Ratcliff, 1979). After pooling (outliers had been excluded), the numbers of trials for the two frequent-response conditions were 2836 and 2844 in Experiment 1, 3782 and 3782 in Experiment 2, and 3248 and 3213 in Experiment 3; for the two infrequent-response conditions they were 952 and 950 in Experiment 1, 1268 and 1267 in Experiment 2, and 1070 and 1079 in Experiment 3.

From the pooled data we computed for each condition the mean reaction time of correct responses, the error percentage, and nine quantiles (.1 .2, …, .8, .9) of the reaction-time distribution of correct responses. Models were fit by successively minimizing two measures of the discrepancy between simulated and observed data. The first measure was the square root of the mean weighted squared deviations of the observed error percentages and reaction-time quantiles from the predicted ones. Starting with the parameter estimates obtained with this measure, we then minimized G^2^ computed across the frequencies of errors and correct reaction times in each of the bins defined by the nine quantiles (cf. Ratcliff & Smith, 2004). Details of the fitting procedure are described in Appendix B.

Fitting of a sequential-sampling model requires the estimation of reaction-time distributions in the various experimental conditions given a certain set of model parameters. Whereas for the diffusion model there are different ways of doing so (Richter, Ulrich, & Janczyk, 2023), for our extended LCA models only the least efficient method is available, namely to estimate the distributions by simulating a large number of trials. Even with 100,000 simulated trials per condition, as we used, estimates of the reaction-time distributions are still noisy, and so are the measures of their deviations from the observed distributions. A conspicuous example of such variability of goodness-of-fit criteria is reported by Miletic, Turner, Forstmann, and van Maanen (2017, Fig. 4,5) for log-Likelihoods computed from probability-density approximations to simulated reaction times.

Given the variability of goodness-of-fit criteria, we computed our goodness-of-fit criterion for each model and each experiment 100 times with the estimated parameters, each time with 100,000 simulated trials. For the comparison of the different models with different numbers of free parameters we used the mean AIC with twice the number of the free parameters as a penalty term added to the estimates of G^2^ (cf. Kelly, Corbett, & O’Connor, 2021). However, model selection can be based on different measures. Here we also analyzed the deviations between observations and model simulations in some detail, both for mean reaction times and error rates as well as for the delta plots. Specifically, we estimated the model predictions for reaction times and error rates by way of computing mean reaction times, error percentages and delta plots for 1,000 samples with the same number of trials per condition as in the observed data set. From the distributions of the simulated error rates and reaction times we derived “prediction intervals” in which 95% of samples would fall. These intervals around the means of the simulated data give an indication of the correspondence between simulations and observations for each model.

## 3. Experimental Results

The raw data from the three experiments have been published on the Mendeley Repository (Wühr & Heuer, 2025b).

### 3.1. Experiment 1

Outlier trials were excluded from all analyses of reaction times and errors. Their percentages ranged from 0.6 to 1.4% across the four conditions of Experiment 1.

Mean reaction times were significantly shorter for frequent than for infrequent responses (391 vs 432 ms), *F*(1,15) = 23.63, MSE = 1142.29, *p* < .001, \[\eta _{p}^{2}=.612\]. Similarly, they were shorter in congruent than in incongruent trials (401 vs 423 ms), *F*(1,15) = 28.83, MSE = 267.04, *p* < .001, \[\eta _{p}^{2}=.658\]. Of major interest for the purpose of the present experiments is the modulation of the congruency effect by the relative frequency of responses. This modulation was reflected in a significant interaction of frequency and congruency, *F*(1,15) = 18.51, MSE = 51.21, *p* < .001, \[\eta _{p}^{2}=.552\]. Testing the model including this interaction against a model with only the main effects of frequency and congruency revealed a Bayes factor of B_1_ = 15.3, providing ‘strong evidence’ for the interaction. In an additional analysis we compared the Simon effects at both response frequencies by means of a one-tailed t-test: the mean Simon effects (with standard errors) were 30 (±4.5) ms for frequent responses and 14 (±4.5) ms for infrequent responses, and they were significantly different, *t*(15) = 4.302, *p* < .001, *d* = 1.076, B_1_ = 114.16 (‘extreme evidence’).

**Figure 2:**
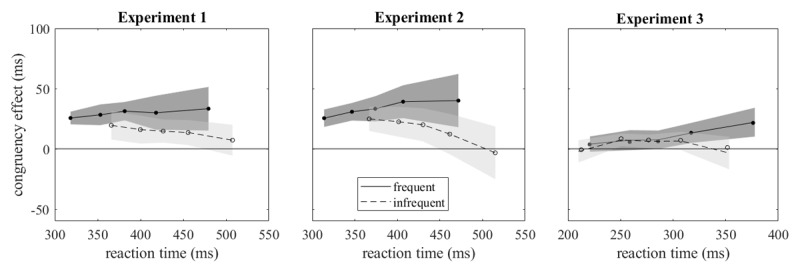
Delta plots shown separately for frequent and infrequent responses. Shaded areas mark the 95% confidence intervals.

The delta plots of Figure 2 (left panel) show the congruency effects as they evolved with increasing reaction times. They were smaller for infrequent than for frequent responses throughout, consistent with the difference observed for mean reaction times, and they tended to diverge, increasing for frequent and decreasing for infrequent responses. The statistical analysis was based on the coefficients of orthogonal polynomials fitted to the individual delta plots. Zero-order coefficients, which characterize the overall level of delta plots, were larger with frequent than with infrequent responses, *t*(15) = 4.153, *p* < .001, *d* = 1.038, B_1_ = 44.0 (‘very strong evidence’). First-order coefficients, which characterize the slopes of the delta plots, were larger with frequent than with infrequent responses, but this difference was only small and statistically not significant, *t*(15) = 1.827, *p* = .088, *d* = 0.457, B_1_ = 1.0 (no evidence in favor of one of the hypotheses). Finally, for second-order coefficients, which characterize the curvature of delta plots, there was no significant effect of relative response frequency, *t*(15) = 0.240, *p* = .814, *d* = 0.060, B_0_ = 3.8 (‘moderate evidence’).

Error percentages were smaller for frequent than for infrequent responses, and the congruency effects for frequent and infrequent responses were 1.9 and 4.4%. As indicated by Wilcoxon signed-rank tests, both congruency effects were significantly different from zero, but the difference between them was not significant, *z* = 0.931, *p* = .375, B_0_ = 2.2.

### 3.2. Experiment 2

Of main interest in this experiment was the effect of the distance of the monitor on the modulation of the Simon effect by relative response frequency. Such an effect of distance was conspicuously absent: with the 50-cm distance of the monitor, the Simon effects were 34 (±4.9) and 15 (±5.9) ms for the frequent and infrequent response, respectively, and with the 150-cm distance they were 34 (±6.3) and 17 (±7.1) ms.

In a three-way ANOVA the relevant interaction of distance, relative frequency, and congruency was not significant, *F*(1,15) = 0.168, MSE = 69.45, *p* = .688, \[\eta _{p}^{2}<.011\]. Testing the model including this three-way interaction against a null model without this interaction provided a Bayes factor of B_0_ = 190.0, indicating ‘extreme evidence’ for the null hypothesis of no contribution of this interaction. The other effects of the distance of the monitor were also nonsignificant in the ANOVA, all *F* < 1. As a further test of an eventual influence of the monitor distance we compared the individual modulations of the Simon effect by relative response frequency between the two distances by means of a t-test, *t*(15) = 0.409, *p* = .688, *d* = 0.102, B_0_ = 3.6 providing ‘moderate evidence’ for the null hypothesis.

For error percentages, the Simon effects with the 50-cm distance of the monitor were 0.6 and 2.9% for the frequent and infrequent responses, respectively, and for the 150-cm distance they were 0.4 and 3.6%. Comparison of the modulations by relative response frequency by means of a Wilcoxon signed-rank test revealed no significant difference, *z* = 0.511, *p* = .629, B_0_ = 3.3 (‘moderate evidence’ for the null hypothesis).

All further analyses of the results of Experiment 2 were run with the collapsed data obtained with the two monitor distances. Outlier percentages ranged from 0.3 to 1.7% across the four conditions. Reaction times were significantly shorter for frequent than for infrequent responses (384 vs 436 ms), *F*(1,15) = 47.46, MSE = 927.91, *p* < .001, \[\eta _{p}^{2}=.760\], and they were shorter in congruent than in incongruent trials (398 vs 423 ms), *F*(1,15) = 28.68, MSE = 341.14, *p* < .001, \[\eta _{p}^{2}=.657\]. Crucially the interaction of frequency and congruency was significant, *F*(1,15) = 7.52, MSE = 197.18, *p* < .015, \[\eta _{p}^{2}=.334\]. Testing the model including this interaction against a model with only the main effects of frequency and congruency revealed a Bayes factor of B_1_ = 3.6, providing ‘moderate evidence’ for the interaction. In an additional analysis we compared the Simon effects at both response frequencies by means of a one-tailed t-test: the mean Simon effects were 34 (±5.2) ms for frequent and 15 (±6.3) ms for infrequent responses, and they were significantly different, *t*(15) = 2.743, *p* = .008, *d* = 0.686, B_1_ = 7.7 (‘moderate evidence’).

The delta plots are shown in Figure 2 (central panel). As in Experiment 1, they were consistently smaller for infrequent than for frequent responses and tended to diverge, increasing for frequent and decreasing for infrequent responses. Zero-order coefficients, which characterize the overall level of delta plots, were significantly larger with frequent than with infrequent responses, *t*(15) = 2.605, *p* = .020, *d* = 0.651, B_1_ = 3.1 (‘moderate evidence’). First-order coefficients, which characterize the slopes of the delta plots, were also larger with frequent than with infrequent responses, *t*(15) = 3.611, *p* = .003, *d* = 0.903, B_1_ = 17.1 (‘strong evidence’). Finally, second-order coefficients, which characterize the curvature of delta plots, were not significantly different between relative response frequencies, *t*(15) = 0.570, *p* = .577, *d* = 0.142, B_0_ = 3.4 (‘moderate evidence’ for no difference between curvatures).

Error percentages were smaller for frequent than for infrequent responses (0.7 vs 6.1%). The congruency effects for frequent and infrequent responses were 0.5 and 3.3%. As indicated by Wilcoxon signed-rank tests, only the larger of these congruency effects was significantly different from zero, and the difference between them was significant, *z* = 2.158, *p* = .033, B_1_ = 3.9.

### 3.3. Experiment 3

Outlier percentages ranged from 6.0 to 7.1% across the four conditions. The higher proportions of outliers than in Experiments 1 and 2 were primarily due to premature responses (without them outlier percentages ranged from 0.6 to 0.9%).

The findings of Experiment 3 differed from those of Experiments 1 and 2. Reaction times were longer rather than shorter for frequent than for infrequent responses (294 vs 280 ms), *F*(1,23) = 25.15, MSE = 181.57, *p* < .001, \[\eta _{p}^{2}=0.522\]. In congruent trials reaction times were shorter than in incongruent trials (283 vs 291 ms), *F*(1,23) = 14.71, MSE = 100.38, *p* < .001, \[\eta _{p}^{2}=0.243\], indicating the presence of a small Simon effect. Crucially, the interaction of frequency and congruency was not significant, *F*(1,23) = 2.02, MSE = 131.07, *p* = .168, \[\eta _{p}^{2}=0.081\]. Testing the full model against a null model without the interaction revealed a Bayes factor of B_0_ = 1.22 (‘anecdotal evidence’ for no effect of relative response frequency on the Simon effect). In an additional analysis the mean Simon effects of 11 (±3.4) ms and 5 (±2.8) ms for frequent and infrequent responses were not significantly different by a one-tailed t-test, *t*(23) = 1.422, *p* = .084, *d* = 0.314, B_0_ = 1.06 (‘anecdotal evidence’ for the hypothesis of no difference).

The modulation of the Simon effect by relative response frequency amounted to only 6.6 (±4.7) ms in this experiment as compared with 17.3 (±3.9) ms in Experiments 1 and 2 (collapsed). This difference was significant by a one-tailed t-test, *t*(54) = 1.766, *p* = .042, *d* = 0.477, B_1_ = 1.85 (‘anecdotal evidence’). Thus, overall there is no evidence of a modulation of the Simon effect by relative response frequency in Experiment 3, though the evidence for the absence of such a modulation (and also a weaker modulation than in Experiments 1 and 2) is only weak.

The delta plots of Figure 2 (right panel) show a small difference between frequent and infrequent responses which emerged only at the longest reaction times. Zero-order coefficients, which characterize the overall level of delta plots, were not significantly different between frequent and infrequent responses, *t*(23) = 1.793, *p* = .086, *d* = 0.366, B_0_ = 1.2 (‘anecdotal evidence’ for no difference), but first-order coefficients were larger with frequent than with infrequent responses, *t*(23) = 2.291, *p* = .031, *d* = 0.468, B_1_ = 1.9 (‘anecdotal evidence’). Similarly, second-order coefficients, which characterize the curvature of delta plots, were significantly different, *t*(23) = 2.914, *p* = .008, *d* = 0.595, B_1_ = 6.0 (‘moderate evidence’): delta plots had upward curvature for frequent responses and downward curvature for infrequent responses.

Error percentages were slightly smaller for frequent than for infrequent responses (0.6 vs 1.6%), and congruency effects were 0.8% both for frequent and infrequent responses (Wilcoxon signed-rank test: *z* = 0.017, B_0_ = 4.7).

## 4. Model-based analyses

The evaluation of different computational models is typically based on a measure of goodness-of-fit (cf. Busemeyer & Diederich, 2014). However, there are different measures, and these are differently affected by different deviations between observations and predictions. Therefore, we focus not only on an overall measure of goodness-of-fit, but also in some detail on the agreement of the simulated data with the observed ones and the consistency of parameter differences with the hypotheses the models were designed to implement.

**Figure 3:**
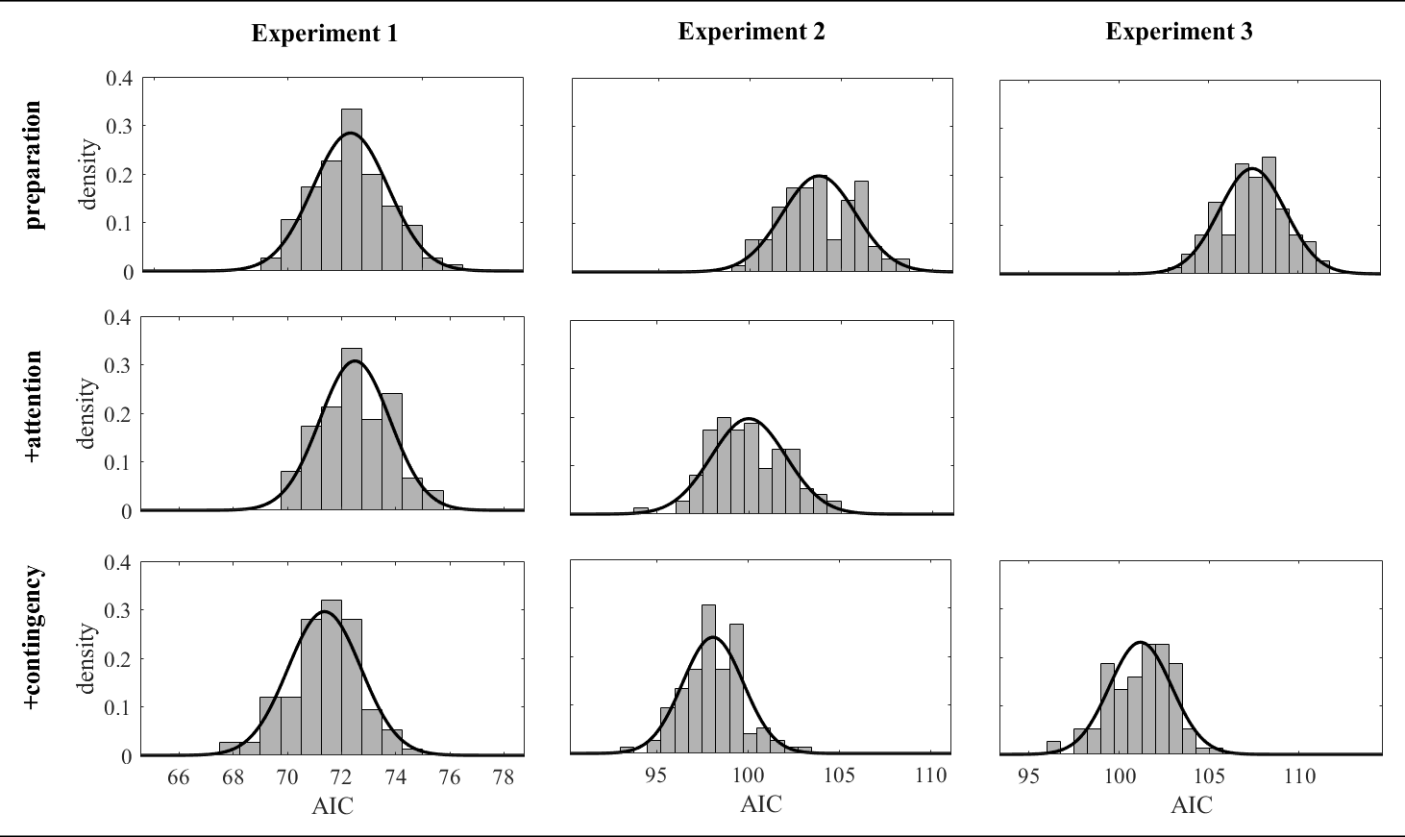
Distributions of the AIC for the three models as fitted to the three experiments (the +attention model was not fitted to Experiment 3).

Our first step of model evaluation is based on the AIC as estimated from G^2^ with twice the number of free parameters as penalty term. With simulated reaction-time distributions, as for the present models, goodness-of-fit measures are noisy. Figure 3 shows the distributions of the AIC for each model and each experiment. Each distribution is based on 100 simulations of 100,000 trials each per condition and approximated by a normal distribution; according to Shapiro-Wilk tests, for no model and no experiment the distribution of the sample of 100 simulated AIC deviated from the normal. The means and standard deviations of the AIC are shown in Table 2: in terms of the AIC, there was only a very slight advantage of the +contingency model over the preparation model for Experiment 1, but stronger advantages for Experiments 2 and 3. The +attention model had an advantage over the preparation model only in Experiment 2.

**Table 2:** Means and standard deviations of the AIC for the three models as fitted to the three experiments.


	EXPERIMENT 1		EXPERIMENT 2		EXPERIMENT 3	
		
MODEL	MEAN	s	MEAN	s	MEAN	s

preparation	72.3	1.40	103.8	2.01	107.5	1.83

+ attention	72.5	1.29	100.0	2.03	–	–

+ contingency	71.4	1.34	98.1	1.66	101.2	1.72


**Figure 4:**
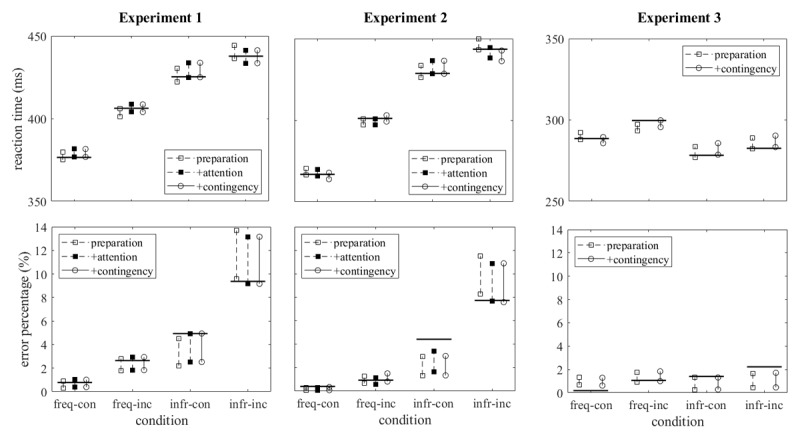
Observed and predicted mean reaction times and error rates. Observed data are indicated by the horizontal lines for each of the four conditions, predicted data by the predictions interval (95% intervals of the distributions of the simulated mean reaction times and error rates obtained with 1000 runs, each one with the same number of simulated trials per condition as in the actual sample). For Experiments 1 and 2 predicted data are shown for three models as indicated in the insets, for Experiment 3 the +attention model was not fitted.

Our second step in model evaluation is the examination of the agreement between simulated and observed data. In Figure 4 the observed mean reaction times and error rates are compared with the simulated ones. All three models produce simulated mean reaction times which agree rather well with the observed mean reaction times; for none of the models the observed means are clearly outside the prediction intervals. For the mean error percentages, the differences between the three models are also minor, but the agreement with the observed data is poorer. For Experiments 1 and 2 the simulated error rates for infrequent congruent responses tend to be too low and for infrequent incongruent responses too high, and for Experiment 3 the simulated errors rates tend to be too high for frequent and too low for infrequent responses.

**Figure 5:**
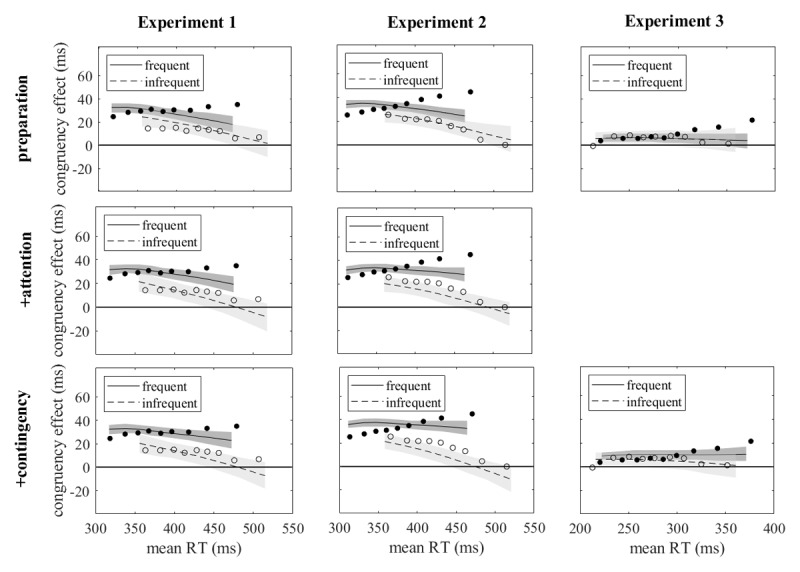
Simulated delta plots with prediction intervals (shaded areas) and observed delta plots (filled and open circles) in all three experiments and for all three models (the +attention model was not fitted to the results of Experiment 3).

Figure 5 shows the simulated delta plots together with the observed ones. Whereas the simulated mean reaction times agreed rather well with the observed means for all three models, this was different for the delta plots. A critical feature of the observed delta plots for frequent and infrequent responses was their divergence with increasing reaction times. This divergence was absent in the predictions of the preparation model (upper row of graphs of Figure 5), hardly visible in the predictions of the +attention model, and more clearly present in the predictions of the +contingency model. However, even for the +contingency model the difference in the slopes of the delta plots was clearly too small for Experiments 1 and 2.

Our third step of model evaluation is the check of the consistency of parameter differences with the hypotheses implemented by the models. For all three models the estimated probability of preparing the frequent response (parameters π in Appendix C) was close to 0.9 for Experiments 1 and 2, and the probability of preparing the infrequent response (1-π) was about 0.1. In Experiment 3 the frequent and the infrequent responses were prepared with almost identical probability (about .850 and .885 for frequent and infrequent responses). The difference in the probabilities of preparing the frequent and infrequent response was sufficient to simulate the modulation of the Simon effect for mean reaction times by relative response frequency: the modulation was present with a strong difference in Experiments 1 and 2, but absent with the essentially negligible difference in Experiment 3.

In addition to the difference in probabilities of preparation, the visual-attention hypothesis holds that the impact of the relevant stimulus feature is stronger for stimuli presented on the side of the prepared response. This hypothesized difference was reflected in the estimates of the parameter ΔI_rel_: .539 vs .502 in Experiment 1, .549 vs .517 in Experiment 2 (Table C2 in the Appendix). However, this extension of the preparation model resulted in hardly any improvement of fit.

The +contingency model holds – in addition to the difference in probabilities of preparation – that the behavioral effect of the irrelevant stimulus feature should be stronger for the stimulus location on the side of the frequent response (frequent-congruent and infrequent-incongruent trials) for which the majority of trials are congruent than for the stimulus location on the side of the infrequent response (frequent-incongruent and infrequent-congruent trials) where the majority of trials are incongruent. A stronger behavioral effect of the irrelevant feature could result from a stronger irrelevant input or a weaker relevant input. In fact, for the strength of the irrelevant input the estimated parameters showed the proper differences in Experiment 1 and 2 (.298 vs .273 and .318 vs .284), but hardly in Experiment 3 (.052 vs .051). However, for the strength of the relevant input the estimated parameters showed the opposite of the expected difference, with a stronger rather than weaker relevant input when the stimulus location was on the side of the frequent response (Exp. 1: .533 vs .505; Exp. 2: .535 vs .494; Exp. 3: .491 vs .465). These differences between parameter estimates suggest that the external input overall – both related to relevant and irrelevant stimulus features – was stronger for stimuli presented on the side of the frequent response than for stimuli presented on the side of the infrequent response. Thus, although the simulations of the model approached the observed divergence of delta plots for frequent and infrequent responses in particular for Experiment 3, the parametrization is not consistent with the theoretical foundation of the model.

## 5. An account of divergent delta plots

Both the experimental results and the model-based analyses provide no evidence in favor of the visual-attention hypothesis and only weak evidence in favor of the contingency hypothesis. In fact, differences in the probabilities of response preparation are sufficient to account for the modulation of the Simon effect by relative response frequency as far as mean reaction times and error percentages are concerned. However, the preparation hypothesis fails conspicuously to account for the divergence of delta plots. For Experiment 3 the contingency hypothesis is consistent with the small Simon effects at long reaction times, but not with the divergent delta plots observed in Experiments 1 and 2.

There is some indication that the divergence of delta plots is not a consequence of relative response frequency per se, but a consequence of different levels of response preparation: Wühr (2006) observed an increasing Simon effect across the quintiles of the reaction-time distributions with valid response cues, but a decreasing Simon effect with neutral cues. Why are these different dynamics of the Simon effect not captured by the preparation model? In this model, preparation of a response is restricted to its anticipatory activation before a stimulus is presented (cf. Miller, 1998). However, preparation of a response might include additional processes. Possibly these serve to adjust the balance between two basic requirements of adaptive behavior, namely to monitor the environment for potentially relevant stimuli and to protect the pursuit of behavioral goals against distracting stimuli (cf. Dreisbach & Goschke, 2004; Miller & Cohen, 2001), in favor of the second requirement.

The human capability to shield actions against distracting influences can hardly be doubted. For example, the extensive literature on the suppression of distractor effects in visual search clearly shows that such suppression is possible (e.g. Van Moorselaar & Slagter, 2020). In fact, even the effects of much more salient distractors than in the Simon task can be voluntarily suppressed (e.g. Heuer & Schubö, 2025). A certain shielding of prepared responses against environmental influences is also indicated by the delays of responses after different responses have been prepared (e.g. Rosenbaum & Kornblum, 1982), and by the increasing failures of stopping a response the more its preparation has been advanced (e.g. Amelang, 1966; Logan, Cowan, & Davis, 1984). Also, the focus of attention on the goal of a movement during motor preparation (Baldauf & Deubel, 2010) can be seen as a shielding of the current action against other environmental influences. Therefore, to account for the divergent delta plots, we considered a *shielding hypothesis* according to which prepared responses are better shielded against influences of irrelevant stimulus features than unprepared responses.

We implemented the shielding hypothesis in the *+shielding model* by allowing the impact of the irrelevant stimulus feature to be initially weaker for prepared than for unprepared responses and, as a kind of flip side, to decline more slowly. A slower decline could be a possible consequence of the initially weaker influence of the irrelevant stimulus feature but also a consequence of weaker inhibitory processes in relation to prepared than to unprepared responses. This model was fitted to the data of Experiments 1 and 2, but not to the data of Experiment 3 because for that experiment both frequent and infrequent responses were prepared with essentially the same relative frequencies.

**Figure 6:**
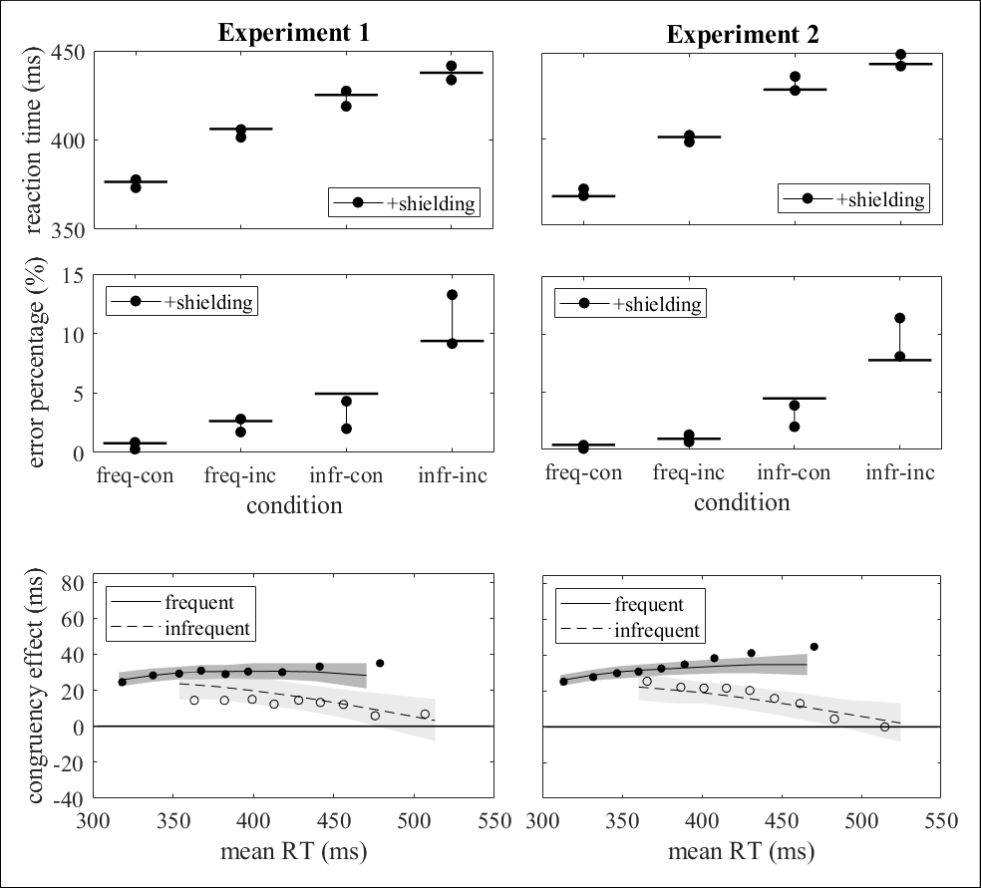
Upper two rows of graphs: Observed and predicted mean reaction times and error rates for the +shielding model in the same format as Figure 4. Lower row of graphs: Simulated delta plots for the +shielding model in the same format as Figure 5.

The mean AIC (with SD) of the model, as estimated from 100 simulations with 100,000 trials each, were 65.2 (1.15) for Experiment 1 and 75.3 (1.52) for Experiment 2. In comparison, the AIC of the +contingency model (cf. Table 2) were 71.4 and 98.1. Thus, the +shielding model reproduced the observed data more accurately than the other models did. As is evident from Figure 6, there was no conspicuous superiority of the +shielding model with respect to mean reaction times and error percentages, but with respect to the delta plots: their observed divergence was rather well, though not perfectly, matched by the simulated delta plots.

## 6. General Discussion

The effect of relative S-R frequency on reaction time and accuracy is a classic result (Fitts, Peterson, & Wolpe, 1963; Teichner & Krebs, 1974), whereas its effect on the size of the Simon effect is a more recent observation: in a binary choice task the Simon effect is stronger for the frequent response with shorter reaction times than for the infrequent response with longer reaction times (Wühr & Heuer, 2015). This modulation of the Simon effect is probably a consequence of the correct or incorrect expectations (and preparations) of responses, as suggested by the following observations. First, the modulation is present when only the relative frequencies of responses differ, but not those of stimuli (Wühr & Heuer, 2015; present experiments). Second, it is also present when equally frequent responses are validly and invalidly cued and thereby expected or unexpected (Proctor, Lu, & Van Zandt, 1992; Verfaellie, Bowers, & Heilman, 1988; Wascher & Wolber, 2004; Wühr, 2006). However, it is unclear whether different expectations per se are crucial or whether additional mechanisms are involved.

In addition to the expectation hypothesis, we envisaged two hypotheses on additional mechanisms. The visual-attention hypothesis follows from the observation of Wascher and Wolber (2004) of a facilitated shift of visual attention to the side of an expected and prepared response. It should facilitate responding in frequent-congruent and infrequent-incongruent conditions, thereby increasing the Simon effect for frequent responses and reducing it for infrequent responses. In the experiments of Wascher and Wolber expectations were induced by response cues presented on the monitor where the stimuli were presented, and these cues rather than response preparations might have been crucial for the facilitated shifts of visual attention. Thus, it is not obvious that facilitated shifts of visual attention would also contribute to the modulation of the Simon effect by relative response frequency.

The contingency hypothesis posits certain consequences of the different relative frequencies per se rather than of the accompanying differences in response preparation. In a Simon task with different response frequencies the irrelevant stimulus locations are associated with different proportions of congruent and incongruent responses. Such a difference typically results in different influences of irrelevant stimulus features (Schmidt, 2013, 2019), and this modulation of the Simon effect can be specific for stimulus locations (Hübner & Mishra, 2016). According to the contingency hypothesis, there should be a stronger facilitating effect of the congruent irrelevant stimulus location for frequent than for infrequent responses, but a weaker inhibitory effect of the incongruent stimulus location. As far as the differences between congruent and incongruent conditions are concerned, the facilitating and inhibiting influences of irrelevant stimulus features could cancel each other, but their influences might also be asymmetric and thus produce the observed modulation of the Simon effect.

We arbitrated the three hypotheses both by an experimental and a model-based approach, as we will discuss first. Subsequently we discuss how response preparation reduces the size of the Simon effect as compared with unprepared responses and the implications for other congruency effects. Finally, we turn to the shielding hypothesis and the different dynamics of the Simon effect for expected and unexpected responses.

### 6.1. Arbitrating between hypotheses on the modulation of the Simon effect

After demonstrating the modulation of the Simon effect by relative response frequency in Experiment 1, we put the attention hypothesis to test in Experiment 2. If the shift of visual attention to the left or right side of the monitor were facilitated by preparing a response with the left or right hand in the same way as it was by response cues presented on the monitor in the experiments of Wascher and Wolber (2004), this cross-modal attentional effect should be reduced as the distance between hands and monitor is increased, and so should be the modulation of the Simon effect. However, the increased distance of the monitor had no such effect, putting doubts on the attention hypothesis. The conclusion based on the experimental data was confirmed by the model-based analysis. Allowing for different impacts of the relevant stimulus feature when stimuli were presented on the side of the expected or unexpected response in the +attention model produced no improvement of the goodness-of-fit over the preparation model for Experiment 1 and only a slight improvement for Experiment 2.

The contingency hypothesis was put to test in Experiment 3. With always valid response cues there should be no difference (or only a small one) between the relative frequencies of expecting and preparing the frequent and the infrequent response. In contrast, the contingencies between irrelevant stimulus locations and congruent or incongruent responses were the same as in Experiments 1 and 2. Thus, if different relative frequencies of response expectations were crucial for the modulation of the Simon effect, it should disappear, whereas it should persist if different contingencies were crucial. In Experiment 3 the modulation of the Simon effect was no longer statistically significant, but the evidence for no difference was only weak. In fact, the modulation of the Simon effect by relative response frequency was absent at shorter reaction times, but slightly present at longer reaction times, resulting in delta plots which diverged at the long reaction times. Only the +contingency model reproduced this pattern of results, showing a clearly better fit than the preparation model.

In spite of the superiority of the +contingency model in particular for Experiment 3, but to some extent also for Experiments 1 and 2, this provided no evidence of the contingency hypothesis. Under this hypothesis the irrelevant stimulus location in conditions frequent-congruent and infrequent-incongruent is associated with a higher frequency of congruent than of incongruent responses, whereas the irrelevant stimulus location in conditions frequent-incongruent and infrequent-congruent is associated with a higher frequency of incongruent than of congruent responses. With a higher frequency of congruent responses congruency effects tend to be stronger than with a higher frequency of incongruent responses, which could result from a stronger impact of the irrelevant stimulus feature and/or a weaker impact of the relevant stimulus feature. However, in all three experiments the impact of the relevant stimulus feature was increased rather than decreased when stimuli were presented at a location associated with more frequent congruent responses. In Experiments 1 and 2 the impact of the irrelevant stimulus feature was increased as well, but in Experiment 3 with very small impact of the irrelevant stimulus feature this was not the case. These parameter estimates are not consistent with the contingency hypothesis. Rather they suggest that stimuli – both their relevant and their irrelevant features – are processed more efficiently when they are presented on the side of the more frequent response.

Both the experimental results and the analysis of the different sequential-sampling models converge on the conclusion that the major part of the modulation of the Simon effect by relative response frequency is a consequence of the different relative frequencies of preparing frequent and infrequent responses. This holds for the mean reaction times and error rates, whereas the different dynamics of the Simon effects for frequent and infrequent responses, as reflected in the divergent delta plots, have other origins. In addition, there is some more tentative evidence that at long reaction times also a difference between processing stimuli at the two different irrelevant locations of a Simon task comes into play, a more efficient processing at the locations on the side of the more frequent response (associated with more congruent responses) than at the location on the side of the less frequent response (associated with fewer congruent responses).

### 6.2. Response preparation and the modulation of the Simon effect

There seems to be no intuitively obvious reason for a larger Simon effect with more extensive than with less extensive response preparation. However, a closer examination of the signals of the preparation model gives some clues. These signals are illustrated in Figure 1, neglecting the noise inherent to response activation and the variability of the lead of the irrelevant impact, but with the other parameters similar to the ones estimated by fitting the preparation model to the observed data. Two aspects of the model parameters seem to be critical for the modulation of the Simon effect. First, the mean lead of the impact of the irrelevant input is similar to its time constant, that is, to the time when the irrelevant input crosses zero. Thus, the delayed impact of the relevant stimulus feature is slightly facilitated by the leading irrelevant stimulus feature in incongruent conditions (rather than inhibited) and slightly inhibited (rather than facilitated) in congruent conditions. As a consequence, when the relevant input becomes effective, the activations of the correct response code in congruent and incongruent conditions converge with the passage of time rather than diverge (what they did before the relevant input was processed). Second, the initial level of response code activations is smaller for unprepared responses so that the convergence of response code activations in congruent and incongruent conditions extends over a longer time, resulting in smaller reaction-time differences.

Although there could perhaps be other ways of how response preparation could produce a modulation of the Simon effect, the above analysis suggests certain boundary conditions. First, the impact of the irrelevant stimulus feature should be reversed for some time, as it is also posited by the Diffusion Model for Conflict tasks (Ulrich et al., 2015). Second, there should be a lead of the impact of the irrelevant stimulus feature as it is typical for the Simon task (Cespon et al., 2020; Zorzi & Umiltá, 1995), but not for the Eriksen flanker task with letters as target (cf. Heuer & Wühr, 2025) or for a task in which color is the relevant and stimulus size the irrelevant stimulus feature (cf. Wühr & Heuer, 2025a). Given these differences between conflict tasks, which result in systematically different delta plots for the respective congruency effects, the present findings could be specific for the Simon task and not generalize to other conflict tasks, specifically not to conflict tasks without a lead of the impact of the irrelevant stimulus feature or even a lag. At present there is only some suggestive evidence: the congruency effect in the Eriksen flanker task, for which there is probably no or only a small systematic temporal offset between relevant and irrelevant stimulus features, was found to be of the same size with valid and invalid response cues (Wühr & Heuer, 2017).

### 6.3. The shielding hypothesis

The preparation hypothesis, as implemented by the preparation model, accounted well for the modulation of the Simon effect by relative response frequency as far as mean reaction times and error rates were concerned, but failed to account for the divergent delta plots. These divergent delta plots were rather well simulated by the +shielding model, which implemented the shielding hypothesis for the prepared responses.

The notion that prepared responses become progressively shielded against distracting influences until the “point of no return” (Logan, 2015; Schultze-Kraft, Birman, Rusconi, Allefeld, Görgen, Dähne, Blankertz, & Haynes, 2016) may appear to be at variance with the experimental findings because the effect of the distracting stimulus location, and thus the Simon effect, is actually stronger for prepared than for unprepared responses. However, shielding is part of preparatory processes so that it is the initial impact of the irrelevant stimulus location that is reduced. A kind of flip side of this preparatory shielding is a slower decline of the impact of the irrelevant stimulus feature. Perhaps the initially weaker impact of the irrelevant feature triggers only weaker inhibition, or inhibitory processes related to a prospective movement may be weakened as preparation proceeds, similar to the capability to inhibit the overt response. The slower decline of the irrelevant input can result in a stronger behavioral effect of the irrelevant stimulus feature in spite of its initially weaker effect, in particular when the decline of the stronger irrelevant input largely occurs before the impact of the relevant input as in the present experiments.

We implemented the shielding against distraction for prepared responses because divergent delta plots have also been reported for equally frequent, but cued and uncued responses (Wühr, 2006). However, for Experiments 1 and 2 prepared responses were almost always the frequent ones and unprepared responses the infrequent ones. Therefore, the divergent delta plots can also be simulated when frequent instead of prepared responses are shielded against distraction. At present, however, the balance seems to favor response preparation as the process that includes shielding against distraction.

### 6.4. Conclusions

In a binary choice task with different response frequencies the Simon effect is larger for the frequent response than for the infrequent one. This difference is a consequence of more frequent correct expectations and preparations of the frequent than of the infrequent response, where response preparation probably includes shielding against distraction (with a slower decline of the remaining small distraction later on) in addition to anticipatory response activation. When the differences between frequent and infrequent responses with respect to expectations are eliminated by way of always valid response cues, a small Simon effect remains at long reaction times which probably results from more efficient processing of stimuli presented on the side of the frequent response.

## Data Accessibility Statement

During the reviewing process, the raw data from the experiments can be obtained from the second author upon request. The raw data from the three experiments have been published on the Mendeley repository (Wühr & Heuer, 2025b).

## Code Availability

The programs, which were used for performing model simulations, can be obtained from the first author upon request.

## Additional File

The additional file for this article can be found as follows:

10.5334/joc.471.s1 Appendices. Appendix A to C.

## Footnotes

We are grateful to an anonymous reviewer of a previous version of the manuscript who has drawn our attention to this.

Experiments 2 and 3 were conducted in 2015 and 2016, respectively. Although these experiments are not new, they had been designed for testing the same, or very similar, hypotheses as those described.
