# Supplementary material for: Response Preparation and the Simon Effect: Experimental and Model-Based Analyses
Source: J Cogn. 2026 Jan 7;9(1):1. doi: 10.5334/joc.471 (PMC12785706; doi:10.5334/joc.471)
Supplement: Appendices. — Appendix A to C. [file joc-9-1-471-s1.pdf]

Supplementary Materials to Heuer & Wühr (2025)

Appendix A

All models used in this study were extensions of the Leaky-Competing Accumulator (LCA) model proposed by Usher and McClelland (2001). These extensions were decompositions of the external input, which we have described previously (Wühr & Heuer, 2018; Heuer et al., 2023, Heuer & Wühr, 2025; Wühr & Heuer, 2025), and the introduction of a probability of preparing the correct response.

According to Usher and McClelland (2001, Eq. 3), the instantaneous activations $\Delta a_c(i)$ and $\Delta a_e(i)$ of response codes for correct and incorrect responses, respectively, during each time interval i are given by:

(1a) $\Delta a_c(i) = [\, I(i) - \lambda\, a_c(i) - \beta\, a_e(i)]\, (\Delta t/\tau) + \xi(i) \sqrt{}\, (\Delta t/\tau)$

(1b) $\Delta a_e(i) = [\, (1\text{-}I(i)) - \lambda\, a_e(i) - \beta\, a_c(i)]\, (\Delta t/\tau) + \xi(i) \sqrt{}\, (\Delta t/\tau)$

with self-inhibition gain λ, lateral-inhibition gain β, and Gaussian noise $\xi(i)$ with zero mean and standard deviation σ_n. The external inputs I(i) and 1-I(i) add to 1 (cf., Usher & McClelland, 2001, p.559). As a default we set I(i) = 0.5, so that they are identical for the correct- and error-response codes, and specify external inputs as additions to 0.5. The constraint on the sum of the external inputs results in a forward inhibition of the error-response code in addition to the lateral inhibition when I(i) >1 and thus 1-I(i)<0.

The instantaneous activations of each response code are cumulated, beginning at initial values $a_c(0)$ and $a_e(0)$, and the cumulated activation of each code is bound to be non-negative:

(2a) $a_c(i) = \max\, [\, 0, a_c(i\text{-}1) + \Delta a_c(i)]$

(2b) $a_e(i) = \max\, [\, 0, a_e(i\text{-}1) + \Delta a_e(i)]$.

When the activation of one of the two response codes, $a_c(i)$ or $a_e(i)$, reaches a threshold θ, the respective correct or error response is initiated. A non-decision or residual time R is added to the time needed for the decision.

Previously (Wühr & Heuer, 2018; Heuer et al., 2023) we have extended the basic LCA model by the addition of a declining external input related to the task-irrelevant stimulus feature. It is designated as irrelevant input in contrast to the relevant input. A unique feature of the model is a variable temporal offset between relevant and irrelevant input. Thus we define the external input I(i) as the sum of two components (added to the default of 0.5): the time-invariant relevant input ΔI_{rel} and the time-varying irrelevant input $g(t)*\Delta I_{irr}$. Previously we defined g(t) as the step response of a first-order high-pass filter, that is, as exponential decline from 1 to 0 with time constant δ, $g(t) = e^{-t/\delta}$. However, recently we found a better fit with the step-response of a second-order high-pass filter, that is, a decline to zero that becomes negative on the way, specifically at time $t = \delta$, $g(t) = (1 - t/\delta) * e^{-t/\delta}$ (Wühr & Heuer, 2025). This is the type of decline which we use here as well.

For the temporal offset *D* between the relevant and irrelevant inputs we define a uniform distribution with mean μ_D and width w_D. The time at which the relevant input becomes available is t = 0; with a sample value of $d < 0$ the irrelevant input leads as it is typical for the Simon task with horizontally arranged stimulus and response locations, and with a $d>0$ the relevant input leads. The decline of g(t) thus starts at time *d* rather than 0. For $d < 0$, that is, with leading irrelevant input, the external input is

(3a) $$I(t) = \begin{cases} 0.5 + g(t) * \Delta I_{irr} & \text{for } t < 0 \\ 0.5 + \Delta I_{rel} + g(t) * \Delta I_{irr} & \text{for } t \geq 0 \end{cases}$$

with $g(t) = (1 - (t - d)/\delta) * e^{-(t-d)/\delta}$, and for $d > 0$, that is, with leading relevant input, it is

(3b) $$I(t) = \begin{cases} 0.5 + \Delta I_{rel} & \text{for } 0 \leq t \leq d \\ 0.5 + \Delta I_{rel} + g(t) * \Delta I_{irr} & \text{for } t > d \end{cases}$$

Accumulation of the instantaneous activations starts as soon as the relevant or irrelevant input becomes available, whatever is earlier, that is at min(0,d). Simulated reaction time is the time from the start of accumulation until the threshold θ is reached plus the residual time *R*.

To account for the effects of relative response frequency in Experiments 1 and 2, we introduce a parameter π which specifies the proportion of frequent responses that are expected

(and prepared), with 1-π being the proportion of infrequent responses expected. The bias parameter *B* defines the initial activation of the expected response: when the correct response is expected, $a_c(0) = B$ and $a_e(0) = 0$, but when the error response is expected, $a_c(0) = 0$ and $a_e(0) = B$. This is the only difference between expected and unexpected responses. In Experiment 3 expectations of frequent and infrequent responses were essentially made identical by means of response cues. Thus, probabilities of expecting the one or the other response were no longer π and 1-π, but π_{freq} and π_{infr}, allowing a possible influence of response frequency on expectations in addition to the effect of always valid response cues.

For simulations of the models we set $\Delta t = 0.001$ and $\tau = 0.1$, so that $\Delta t/\tau = 0.01$. For each cycle i of the simulations, activations of the response codes were updated preliminarily based on the external inputs I(i) and 1-I(i) and the noise $\xi(i)$, and thereafter the preliminary updates of the response-code activations were used to compute self-inhibitions and lateral inhibitions for the instantaneous activations which then served to finally update the response-code activations.

The model parameters are listed in Table A1. It should be noted that the four conditions, to which the models were fitted simultaneously, differed in only a few parameters: Congruent and incongruent conditions differed only in the arithmetic sign of ΔI_{irr}, being positive for congruent and negative for incongruent conditions. Frequent and infrequent responses differed only in the probability of expecting the correct response, which were π and 1-π, respectively, for Experiments 1 and 2, but π_{freq} and π_{infr} for Experiment 3. For Experiment 3 we also allowed a difference in the residual time *R* to take the rather unexpectedly faster infrequent than frequent responses into account.

In addition to the preparation model we tested extensions which allowed for additional differences between frequent and infrequent responses. The ‘+attention’ model allowed different parameters ΔI_{rel} for locations that are congruent with the side of the expected response, which according to the visual-attention hypothesis should facilitate the shift of

visual attention to that location, and for locations which are incongruent with the side of the expected response. The '+contingency' model allows different parameters ΔI_{rel} and ΔI_{irr} depending on whether the irrelevant stimulus location is congruent with the frequent response – this location is associated with a majority of congruent trials – or incongruent – that location is associated with a majority of incongruent trials. Specifically these are the conditions frequent-congruent and infrequent-incongruent on the one hand and frequent-incongruent and infrequent-congruent on the other hand. Finally, the '+shielding' allows different parameters ΔI_{irr} and δ for prepared and unprepared responses.

Table A1: Model parameters (time parameters are in seconds, indicated by s)

λ	self-inhibition gain
β	lateral-inhibition gain
σ_n	standard deviation of noise
π	probability of expecting the frequent response
B	expectancy-induced response bias
ΔI_{rel}	relevant external input
ΔI_{irr}	(automatic) irrelevant external input
δ (s)	time constant for decline of irrelevant input (s)
μ_D (s)	mean temporal offset (s) between relevant and irrelevant input
w_D (s)	width of temporal-offset distribution (s)
θ	response threshold
R (s)	residual time (s)

Appendix B

We fitted models to the four experimental conditions of each experiment. Reaction times of errors were neglected because error frequencies were quite small in some conditions and mean error reaction times therefore quite unreliable. Parameters were estimated in a two-step procedure. In the first step we minimized the square root of the mean weighted sum of the squared deviations between predicted and observed relative error frequencies (in percent) and nine quantiles of the distributions of the pooled reaction times (in seconds) of correct responses (wRMSE):

$$\text{wRMSE} = 1000\sqrt{\frac{1}{4*8.75}\ A} \quad \text{with}$$

$$A = \sum_{j=1}^{4}\left[c_0\left(p_{ob.j} - p_{pr.j}\right)^2 + \sum_{k=1}^{9} c_k\left(P_{ob.kj} - P_{pr.kj}\right)^2\right],$$

where $j = 1, \ldots, 4$ are the four conditions of each experiment, p is the error probability, P_k are the 9 quantiles of the pooled distributions of reaction times of correct responses, and c_k are the weights (.5, 1, 1, 1, 1, 1, 1, 1, .75, .5) for the error probability and the quantiles in increasing order (8.75 is the sum of the weights for each of the four conditions fitted simultaneously). This weighting is similar to weightings used previously (e.g. Heuer et al., 2023), giving less weight to less reliable measures. Subscripts *ob* and *pr* indicate the observed and predicted data, respectively. Multiplication by 1000 improves readability. Minimizing wRMSE served to produce full overlap between simulated and observed reaction-time distributions and thereby to prevent predicted frequencies of zero which would result in infinite values in computing G^2.

In the second step we minimized G^2 (cf. Ratcliff & Smith, 2004), beginning with the parameter estimates obtained with minimizing wRMSE:

$$G^2 = 2\sum_{j=1}^{4}\frac{f_{ob.j}}{f_{ob}}\ \sum_{k=1}^{11} f_{ob.kj} * ln\left[\frac{f_{ob.kj}}{f_{pr.kj}}\right],$$

with f_{ob} being the total number of observation, $f_{ob.j}$ the number of observations in condition j, $j = 1, \ldots, 4$, and $f_{ob.jk}$, $k = 1, \ldots, 11$, the number of errors and correct reaction times in the 10 bins

defined by the 9 quantiles in condition j. For each condition j, $\sum_{k=1}^{11} f_{pr.kj} = f_{ob.j}$, that is, for each condition the sums of observed and predicted frequencies were identical. The parameters estimated by minimization of G^2 are listed in Appendix C.

We started each minimization of wRMSE with 1000 simulated trials per condition, which were increased up to 100,000 trials, using the MATLAB function *fminsearch*. The initial parameter values at the start of the minimizations were set somewhat intuitively to be not too far away from the final estimates. We used successive runs of the function with 75 iterations. The search ended when with 100,000 simulated trials per condition a criterion was reached that included changes of the parameters and the function value (parameters of *fminsearch* were TolX=0.3 and TolFun=0.15). Using the estimated parameters, we subsequently minimized G^2 with 100,000 simulated trials per condition until in one of the runs of 75 iterations the stop criterion was reached (with TolFun=4.0). For all models, except for the post-hoc +shielding model, that allowed different estimates of ΔI_{rel}, ΔI_{irr} or R the initial values of the possibly different estimates were identical. For the +shielding model we shortened the search for a minimum of G^2 by selecting initial parameters consistent with the hypothesis (for expected responses ΔI_{irr} was set about half its size for unexpected responses, and δ about twice its size for unexpected responses).

When predicted reaction-time distributions are estimated by way of simulations, these estimates are noisy, and so are measures of goodness-of-fit. Therefore, we repeated the computation of these measures 100 times for each experiment and each model with the estimated parameters and 100,000 simulations. Model selection was based on the mean AIC, estimated as mean G^2+2m, with m being the number of free parameters. The predicted error percentages, mean reaction times, and delta plots (and the distributions of these estimates) were computed by 1000 simulations with the estimated parameters and the number of trials being the same as the number of observed trials in each condition of each experiment.

Appendix C

Tables C1, C2, C3, and C4 list the parameter estimates as they were obtained by minimizing G^2 as a measure of the deviation of the simulated from the observed data. Although some of the parametric variations are obviously meaningful, such as the weaker impact of the irrelevant input in Experiment 3 than in Experiments 1 and 2, we refrain from a more detailed interpretation for various reasons. The first one is that the parameter estimates are noisy for the same reasons as the goodness-of-fit criteria are. Thus, differences may exist by chance. Second, searching for minima of a multidimensional surface can easily end in local minima or in minima with very shallow gradients so that, given the noise in the estimates of probability density functions, there are regions where the variation of parameters results in only small variations of the goodness-of-fit criterion that are masked by noise. Third, some parameters may have very little effect on the predictions so that their estimates tend to remain close to their initial values. Thus, we report the parameter estimates for the sake of completeness without placing too much weight on them. There is one exception, however: the hypotheses implemented in the +attention, the +contingency, and the +shielding models required certain differences between the parameters, and when these were not present even a better fit of the model was not taken as evidence for the implemented hypothesis.

Table C1: Parameter estimates for the preparation model. Parameters are explained in Table A1; time parameters are in seconds (s).

Parameter	Exp. 1	Exp. 2	Exp. 3
λ	0.215	0.223	0.259
β	0.284	0.275	0.360
σ_n	0.274	0.265	0.182
$^1\pi_{freq}$	0.870	0.936	0.852
π_{infr}	-	-	0.885
B	0.451	0.476	0.486
ΔI_{rel}	0.511	0.508	0.481
ΔI_{irr}	0.291	0.299	0.043
δ (s)	0.111	0.118	0.120
μ_D (s)	-0.117	-0.113	-0.120
w_D (s)	0.134	0.136	0.159
θ	1.327	1.368	1.148
$^2R_{freq}$ (s)	0.192	0.185	0.108
R_{infr} (s)	-	-	0.101

[1]: For Exp. 1 and 2 is $\pi_{infr} = 1 - \pi_{freq}$, only for Exp. 3 these parameters do not add to 1.
[2]: For Exp. 1 and 2 is $R_{infr} = R_{freq}$, only for Exp. 3 these parameters can differ.

Table C2: Parameter estimates for the +attention model. Parameters are explained in Table A1; time parameters are in seconds (s).

Parameter	Exp. 1	Exp. 2	Exp. 3
λ	0.214	0.219	-
β	0.273	0.292	-
σ_n	0.278	0.272	-
$^1\pi_{freq}$	0.859	0.947	-
B	0.460	0.471	-
$^2\Delta I_{rel}+$	0.539	0.549	-
$\Delta I_{rel}-$	0.502	0.517	-
ΔI_{irr}	0.281	0.259	-
δ (s)	0.113	0.122	-
μ_D (s)	-0.116	-0.113	-
w_D (s)	0.139	0.143	-
θ	1.337	1.374	-
R (s)	0.194	0.187	-

[1]: For Exp. 1 and 2 is $\pi_{infr} = 1 - \pi_{freq}$,
[2]: $\Delta I_{rel}+$ is the relevant input for trials in which the shift of visual attention should be (relatively) facilitated by a stimulus location on the same side as the expected response, whereas $\Delta I_{rel}-$ is the relevant input for trials in which the shift of visual attention should be (relatively) inhibited by a stimulus location opposite to the side of the expected response.

Table C3: Parameter estimates for the +contingency model. Parameters are explained in Table A1; time parameters are in seconds (s).

Parameter	Exp. 1	Exp. 2	Exp. 3
λ	0.219	0.221	0.263
β	0.264	0.279	0.357
σ_n	0.277	0.267	0.187
[1]π_{freq}	0.874	0.907	0.850
π_{infr}	-	-	0.886
B	0.444	0.485	0.473
[2]$\Delta I_{rel}>$	0.533	0.535	0.491
$\Delta I_{rel}<$	0.505	0.494	0.465
$\Delta I_{irr}>$	0.298	0.318	0.052
$\Delta I_{irr}<$	0.273	0.284	0.051
δ (s)	0.111	0.116	0.129
μ_D (s)	-0.117	-0.109	-0.121
w_D (s)	0.135	0.137	0.156
θ	1.333	1.375	1.144
[3]R_{freq} (s)	0.193	0.188	0.105
R_{infr} (s)	-	-	0.100

[1]: For Exp. 1 and 2 is $\pi_{infr} = 1 - \pi_{freq}$,
[2]: $\Delta I_{rel}>$ and $\Delta I_{irr}>$ are the relevant and irrelevant inputs for stimulus locations on the same side as the more frequent response, for which the proportion of congruent trials is higher than for stimulus locations on the opposite side, where the relevant and irrelevant inputs are $\Delta I_{rel}<$ and $\Delta I_{irr}<$.
[3]: For Exp. 1 and 2 is $R_{infr} = R_{freq}$, only for Exp. 3 these parameters can differ.

Table C4: Parameter estimates for the +shielding model. Parameters are explained in Table A1; time parameters are in seconds (s).

Parameter	Exp. 1	Exp. 2	Exp. 3
λ	0.220	0.219	-
β	0.275	0.294	-
σ_n	0.273	0.274	-
[1]π_{freq}	0.867	0.942	-
π_{infr}	-	-	-
B	0.461	0.474	-
ΔI_{rel}	0.531	0.521	-
[2]$\Delta I_{irr.prep}$	0.135	0.137	-
$\Delta I_{irr.unpr}$	0.285	0.259	-
[3]δ_{prep} (s)	0.229	0.227	-
δ_{unpr} (s)	0.114	0.115	-
μ_D (s)	-0.116	-0.113	-
w_D (s)	0.140	0.144	-
θ	1.332	1.370	-
[1]R_{freq} (s)	0.193	0.187	-
R_{infr} (s)	-	-	-

[1]: For Exp. 1 and 2 is $\pi_{infr} = 1 - \pi_{freq}$ and $R_{infr} = R_{freq}$, only for Exp. 3 these parameters can differ
[2]: $\Delta I_{irr.prep}$ and $\Delta I_{irr.unpr}$ are the irrelevant inputs for prepared and unprepared responses
[3]: δ_{prep} and δ_{unpr} are the time constants for the decline of the irrelevant input for prepared and unprepared responses
